# Deep learning classification of reproductive tissue from ultrasound: sex determination in red abalone (*Haliotis rufescens*)

**DOI:** 10.3389/frai.2026.1794183

**Published:** 2026-05-18

**Authors:** Edwin A. Solares, Anthony Tong, Sohyun Yoo, Welokiheiakeaeloa Gosline-Niheu, Kevin Jacob, Gordon Feliz, Sachin Loecher, Yuan Zhai, Maggie Mah, Sara E. Boles, Ayodeji E. Fagbohun, Jackson A. Gross

**Affiliations:** 1 Department of Computer Science and Engineering, University of California, San Diego, San Diego, CA, United States; 2 Halıcıoǧlu Data Science Institute, University of California, San Diego, San Diego, CA, United States; 3 Department of Computer Science, University of California, Davis, Davis, CA, United States; 4 Bodega Marine Laboratory, University of California, Davis, Bodega Bay, CA, United States; 5 Department of Animal Science, University of California, Davis, Davis, CA, United States

**Keywords:** computer vision, conservation and production aquaculture, endangered species, high-throughput, machine learning, sustainable food production

## Abstract

**Introduction:**

Accurate sex determination is critical for spawning success in both conservation breeding programs and commercial aquaculture, yet non-invasive methods remain limited in abalone species. Traditional approaches rely on visual inspection, which requires substrate detachment, can cause injury, and may induce premature gamete release. Here, we present the first application of machine learning to automate sex classification in red abalone (Haliotis rufescens) using non-invasive ultrasound imaging technology.

**Methods:**

We developed a labeled dataset of 246 high-quality ultrasound images from 44 individuals and benchmarked seven convolutional neural network architectures: VGG16, VGG19, ResNet50, ResNet101, YOLOv8, YOLOv11, and a custom convolutional neural network. Data partitioning by individual identity was essential to prevent artificially inflated accuracy from image leakage across splits.

**Results:**

The YOLOv8 architecture achieved the highest test accuracy of 85.7% (precision: 0.905 male, 0.816 female; recall: 0.845 male, 0.899 female), outperforming both classical architectures and custom models. Interestingly, a custom reverse VGG architecture with decreasing channel depth outperformed standard VGG models, suggesting that early channel compression may help combat ultrasound speckle noise.

**Discussion:**

Feature activation maps confirmed that models learned to attend to gonadal tissue rather than imaging artifacts. We also demonstrate inference on NVIDIA Jetson edge devices, enabling real-time classification suitable for field deployment. This framework establishes the feasibility of automated, non-lethal sex determination for mollusks and lays the groundwork for applications to endangered abalone species where traditional invasive methods are prohibited.

## 1 Introduction

### 1.1 Conservation background

In the 1970s, populations of California abalone (*Haliotis* spp.) began to decline due to historical overharvesting, disease, starvation, and other climate change factors (Gardner et al., 1995; Altstatt et al., 1996; Hobday and Tegner, 2000; Karpov et al., 2000; Hobday et al., 2000; Rogers-Bennett, Laura et al., 2010; Rogers-Bennett et al., 2021; Peters et al., 2024). Due to the dramatic declines in California abalone, all species are recognized as endangered or critically endangered by the International Union for Conservation of Nature (IUCN) (Peters and Rogers-Bennett, 2018, 2020, 2021a,b,c), whereas the black (*H. cracherodii*) and white abalone (*H. sorenseni*) are federally recognized as endangered species by the United States (Federal Register 66[103]; Federal Register 74[9]), with conservation becoming a critical tool for these species. As a result of these declines in abalone, there is currently no legal recreational or commercial abalone fishery along the west coast of the United States (Karpov et al., 2000). Restoration strategies have included the development of the Final Recovery Plan for Black Abalone (NMF, 2020) and the establishment of a White Abalone Captive Breeding Program (Rogers-Bennett et al., 2016). Recovery efforts seek to enhance abalone reproduction using a combination of approaches, including disease and parasite prevention, water quality and temperature management, and the use of non-invasive ultrasonography in conservation and production aquaculture to determine when animals are reproductively competent (Boles et al., 2022, 2023).

Abalone are herbivorous, dioecious (separate sexes) marine snails that release their gametes into the water column for external fertilization, and their gametogenic cycles vary by species, location, temperature, and season (Hahn, 1989). In captive breeding programs, abalone require regular reproductive monitoring to direct spawning activities. Traditionally, this method has relied on visual sexing of individuals, which can be unreliable due to changes in the gonad quality during the reproductive cycle. Traditionally, to visually inspect an abalone's gonad for sex identification, individuals must be detached from their substrate, which can induce the release of immature gametes due to handling stress and reduce spawning success (*personal observation, S. Boles*). Definitive sex determination may be performed using lethal histological examination, but this approach is prohibited for endangered species. To mitigate this stress response, ultrasound imaging technology has been developed as a non-invasive tool to visualize gonadal development and direct spawning activities without disrupting substrate attachment or initiating premature gamete release (Boles et al., 2022; Neylan et al., 2024).

A key component of successful captive propagation is the ability to accurately determine sex and reproductive condition prior to spawning. As broadcast spawners, abalone require coordinated gamete release for fertilization, and missed or mismatched spawning events not only reduce genetic yield but also waste limited hatchery time and resources. Traditional methods such as biopsy, dissection, or induced spawning are invasive, stress-inducing, and often prohibited for some endangered species. In farmed settings, these procedures can reduce animal welfare and operational efficiency as abalone lack external dimorphism, and sex determination via manual manipulation for gonadal assessment is difficult in immature animals. Further complicating sex determination, in non-reproductive abalone, reproductive status is not reliably size-specific. In red abalone, males may begin producing sperm at shell lengths as small as 75 mm, whereas females do not exhibit mature oocytes until approximately 105–130 mm, and individuals larger than 215 mm may exhibit reproductive senescence, including high rates of necrotic oocytes (Rogers-Bennett et al., 2003).

To address these challenges, ultrasound imaging technology has been validated as a non-lethal method to assess reproductive condition in abalone. This technique enables identification of gonadal vs. digestive tissue and provides a repeatable ordinal gonad index score in red abalone (Boles et al., 2022), which has since been applied to endangered abalone species, demonstrating the utility of ultrasonography in captive breeding programs (Boles et al., 2023). While recent studies have proposed refined metrics, such as gonad area and relative average thickness, to improve interpretation (Zou et al., 2025), these methods still rely on manual measurements, are subject to operator bias, and remain labor-intensive. Even with standardized imaging protocols, expert interpretation remains a bottleneck for high-throughput applications.

### 1.2 Machine learning in aquaculture and biological imaging

Recent advances in machine learning (ML), particularly convolutional neural networks (CNNs), have enabled automated classification of complex biological structures from imaging data, including ultrasound. CNNs extract hierarchical features directly from raw images, bypassing the need for handcrafted descriptors and enabling high-accuracy classification even in noisy, real-world datasets. In medical imaging, CNN-based systems have achieved human-level performance on tasks ranging from tumor detection to retinal disease diagnosis (Esteva et al., 2017; Gulshan et al., 2016). In aquaculture settings, CNN-based models have been applied to detect disease symptoms, estimate biomass, and classify sex and ovarian stage in finfish using ultrasonography (Barulin, 2019; Graham et al., 2022). However, applications of ML to marine invertebrates remain rare, limited by anatomical complexity and the scarcity of labeled datasets (Edie et al., 2023).

Abalone present a compelling model for extending these techniques to mollusks. Unlike most gastropods, whose reproductive organs are tucked deep within a spiral shell, the entire internal body of the abalone exists beneath the shell. The gonad/digestive gland complex is accessible to ultrasound imaging from the ventral foot surface. In large red abalone, this allows the entire gonad to be resolved within a single field of view, whereas in smaller individuals the entire soft body beneath the shell can be visualized, making abalone particularly well suited for non-invasive ultrasonography. Prior ML-based sex prediction studies in abalone have used the UCI Abalone Dataset (Nash et al., 1994), a benchmark dataset of physical measurements from *Haliotis rubra* that includes both non-destructive measurements (shell length, diameter, height, whole weight) and measurements that require destructive processing (shucked weight, viscera weight, dried shell weight, and growth ring counts obtained by sectioning the shell) (Barrera-Hernandez et al., 2023). Sex classification on this dataset has proven notably difficult, with reported accuracies typically falling in the 50–55% range and the best reported non-invasive result reaching only 56.9% after extensive polynomial feature engineering (Dabiri, 2025). While these studies demonstrated that ML classifiers can differentiate sex from morphometric features at rates modestly above chance, the inclusion of destructive measurements in the feature set makes the approach incompatible with conservation goals where animals cannot be sacrificed, and the reliance on tabular morphometric data does not leverage the spatial information available in imaging modalities such as ultrasonography.

A practical constraint shared across medical and veterinary ultrasound domains is the limited availability of labeled training data. A scoping review of deep learning classification across medical imaging modalities found that the majority of ultrasound datasets were private, with studies spanning a wide range of dataset sizes including datasets below 1,000 images (Laçi et al., 2025). Lawley et al. (2024) demonstrated that neural network classification of 16 abdominal ultrasound cross-sections from a balanced subset of only 800 images achieved 79.5% accuracy, within 4.4 percentage points of the 83.9% attained by more complex architectures trained on the full dataset of 26,294 images; notably, the authors concluded that dataset size was a more important factor in determining accuracy than network selection. Similarly, Graham et al. (2022) trained CNN models for ovarian development classification in channel catfish (*Ictalurus punctatus*) using 931 ultrasound images and achieved median accuracies exceeding 98% for a binary classification task. These results demonstrate that useful classification accuracy is achievable from small ultrasound datasets when appropriate augmentation, regularization, and data partitioning strategies are employed, though larger datasets consistently improve performance (Lawley et al., 2024). This finding is consistent with our own experience in the present study, and we identify dataset expansion as a priority for future work (Section 4.6). An additional challenge common to ultrasound datasets is image quality variability; recent work has demonstrated that deep learning-based quality assessment can identify artifact-laden or non-informative frames prior to classification (Abdi et al., 2017; Kucharski et al., 2022), an approach we consider a natural extension of the pipeline presented here.

### 1.3 Study objectives

In this study, we present the first demonstration of ML-assisted sex classification in abalone using ultrasound imaging technology. We introduce a fully labeled dataset of red abalone (*Haliotis rufescens*) ultrasound images, a critically endangered species (Peters et al., 2021), and benchmark a suite of CNN architectures; VGG16, VGG19 (Simonyan and Zisserman, 2015), ResNet50, ResNet101 (He et al., 2016), YOLOv8, YOLOv11 (Jocher et al., 2023; Jocher and Qiu, 2024), and custom CNNs trained on confirmed male and female individuals. While our long-term goal is to assess reproductive maturity, the objectives of this study are to establish a high-throughput, non-invasive sexing tool applicable to both conservation and commercial hatchery workflows. Model performance is evaluated using accuracy, precision, and recall, with additional analyses on the effects of animal size and image quality. By integrating ultrasonography with automated image analysis, this work addresses a critical bottleneck in molluscan aquaculture and lays the foundation for future systems capable of linking soft tissue morphology with reproductive outcomes and spawning success.

## 2 Materials and methods

### 2.1 Abalone husbandry and sexing

Red abalone of two different size classes were sourced from The Cultured Abalone Farm (Goleta, CA). Individual animals were housed at the University of California, Davis Bodega Marine Laboratory in ambient oxygenated seawater flow-through conditions in a 9 L clear, polycarbonate tank and fed *ad libitum* a diet of Dulse (*Devaleraea mollis*) and ABKelp^®^ (AlgalMar, Baja California, MEX). Red abalone were divided into two size classes: the small class (n = 41 individuals), which had a mean weight of 80.6 g (SD ± 15.1) and a mean shell length of 77.2 mm (SD ± 3.51), and the large class (n = 34 individuals), which had a mean weight of 259.7 g (SD ± 56.4) and a mean shell length of 110.53 mm (SD ± 15.3). Sex was determined by visual inspection of gonad coloration by The Cultured Abalone Farm management prior to shipment to the UC Davis Bodega Marine Laboratory. In reproductive red abalone, male gonads display a creamy white to tan coloration, whereas female gonads display a dark green coloration (Rogers-Bennett et al., 2004; Hahn, 1989).

### 2.2 Abalone ultrasound imaging protocol

**Figure 1:**
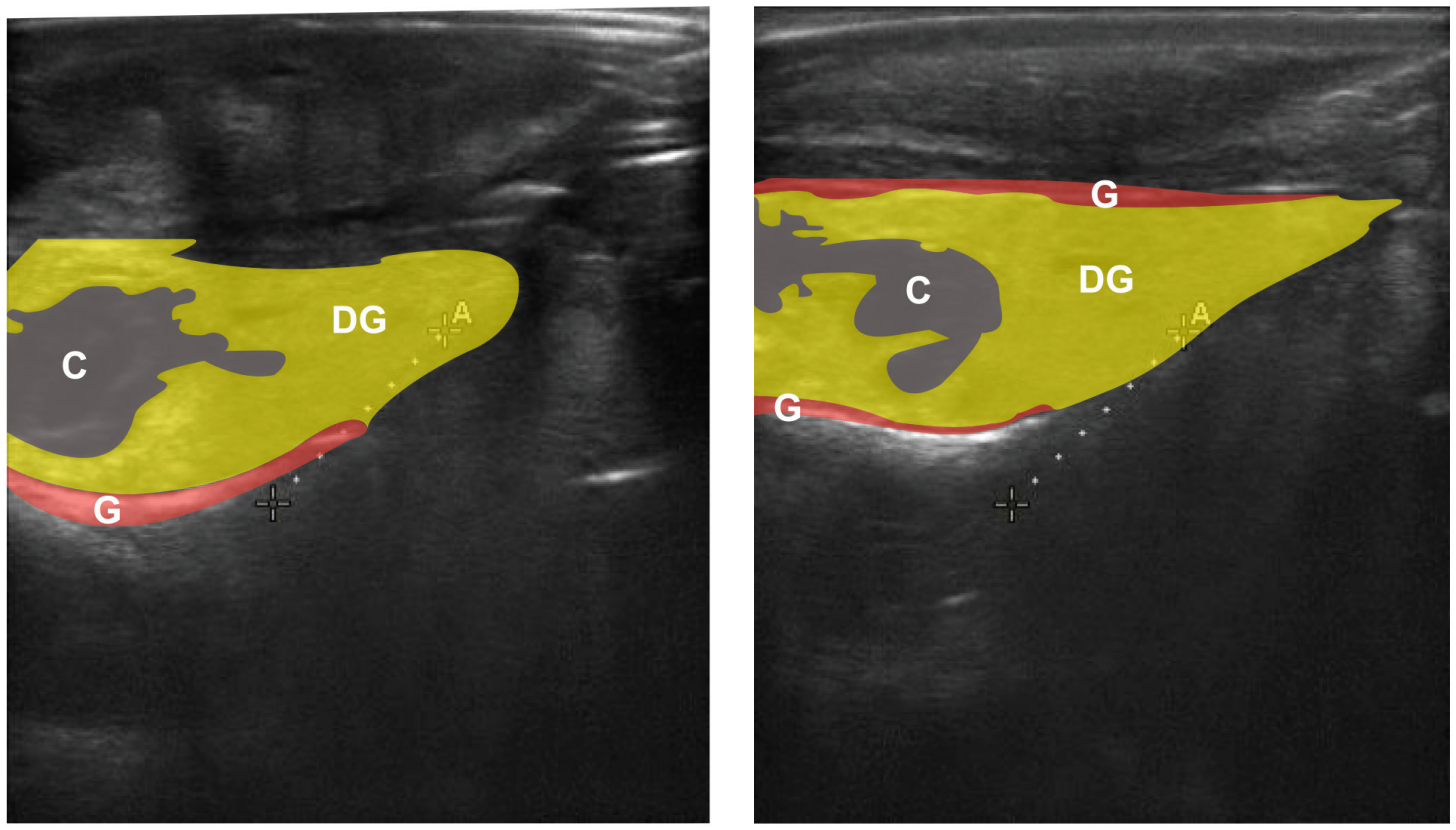
Annotated ultrasound images of red abalone (*Haliotis rufescens*) showing the three principal anatomical structures visible in transverse ultrasonography: coelom (C, blue), digestive gland (DG, yellow), and gonad (G, red). The gonad appears as a thin band enveloping the cone-shaped digestive gland, consistent with the morphology described by (Boles et al. 2022). Two representative large individuals are shown. These annotations were generated manually for illustrative purposes and were not used during model training.

A SonoSite Edge II Ultrasound System (FUJIFILM SonoSite Bothell, Washington) equipped with a HFL50 15-6-MHz transducer probe (Exam Type = Breast; Mechanical Index = 0.7; Read Depth = 6; Thermal Index = 0.1) was used to image red abalone gonad reproductive state. Non-lethal ultrasound examinations were performed by submerging abalone in seawater either attached to clear transparency copier film (3M #PP2950, Austin, Texas) or they remained affixed to their plastic housing (Boles et al., 2022). Red abalone of known sex were examined weekly using ultrasound imaging to characterize sex-specific gonadal architecture, supplemented by analyses of archived images from the same individuals and used as ground truth for training (Figure 1).

### 2.3 Data preparation and augmentation

Ultrasound images of red abalone were preprocessed through an automated pipeline implemented in Python v3.11 (Python Software Foundation, 2023) using Pillow v10.2.0 (Clark, 2015), OpenCV v4.10.0.84 (Bradski, 2000), and NumPy v2.2.2 (Harris et al., 2020). The pipeline consisted of four sequential stages: border removal, region-of-interest (ROI) cropping, filename standardization and organization, zero-padding to uniform dimensions, and dataset partitioning.

#### 2.3.1 Image cropping and organization

Images were first cropped using the Python Pillow library to remove borders and system overlays, which contained metadata including individual identifiers, sex labels, and ultrasound acquisition parameters. Retaining these overlays would risk models learning to classify sex from text rather than from gonadal morphology. Cropped images retained only the raw ultrasonography region. Images were then converted to grayscale and binarized using a fixed intensity threshold of 5 (on a 0–255 scale) to separate the abalone specimen from the dark imaging background. External contours were detected on the binary mask using cv2.findContours with the RETR_EXTERNAL retrieval mode, which identifies only outermost boundaries, and the CHAIN_APPROX_NONE approximation method, which preserves all contour points without simplification. The contour enclosing the largest area was selected as the specimen ROI, and its axis-aligned bounding rectangle was extracted via cv2.boundingRect. The original image was then cropped to this bounding rectangle, yielding specimen-only images of variable dimensions. This approach required no manual annotation; the low threshold value effectively distinguished the specimen from the uniformly dark ultrasound background across all images.

Once cropped, we organized images based on individual (denoted by unique identifiers), sex (Male [M] or Female [F]), and size (small or large). In order to reduce bias in our models we split our data by size, in case there were significant differences in the ultrasound images among the two sizes (Table 1). Unique individuals were identified based on data encoded in the filename string which contained their individual number (ID), location, sex, size, and date. Unique individuals were grouped by their ID, location, sex and size. Images were then deposited into two separate folders (small or large), followed by folders indicating their sex (M or F).

**Table 1:** Distribution of all unfiltered red abalone (*Haliotis rufescens*) images by sex and size class.

	Large	Small	Unknown	Total
F	201 (22)	263 (32)	88 (24)	552 (78)
M	203 (25)	241 (28)	131 (40)	575 (93)
Total	404 (47)	504 (60)	219 (64)	1127 (171)

Unique individual counts are shown in parentheses.

“Unknown” refers to individuals whose size class was not recorded at the time of imaging.

#### 2.3.2 Image quality filtering

**Figure 2:**
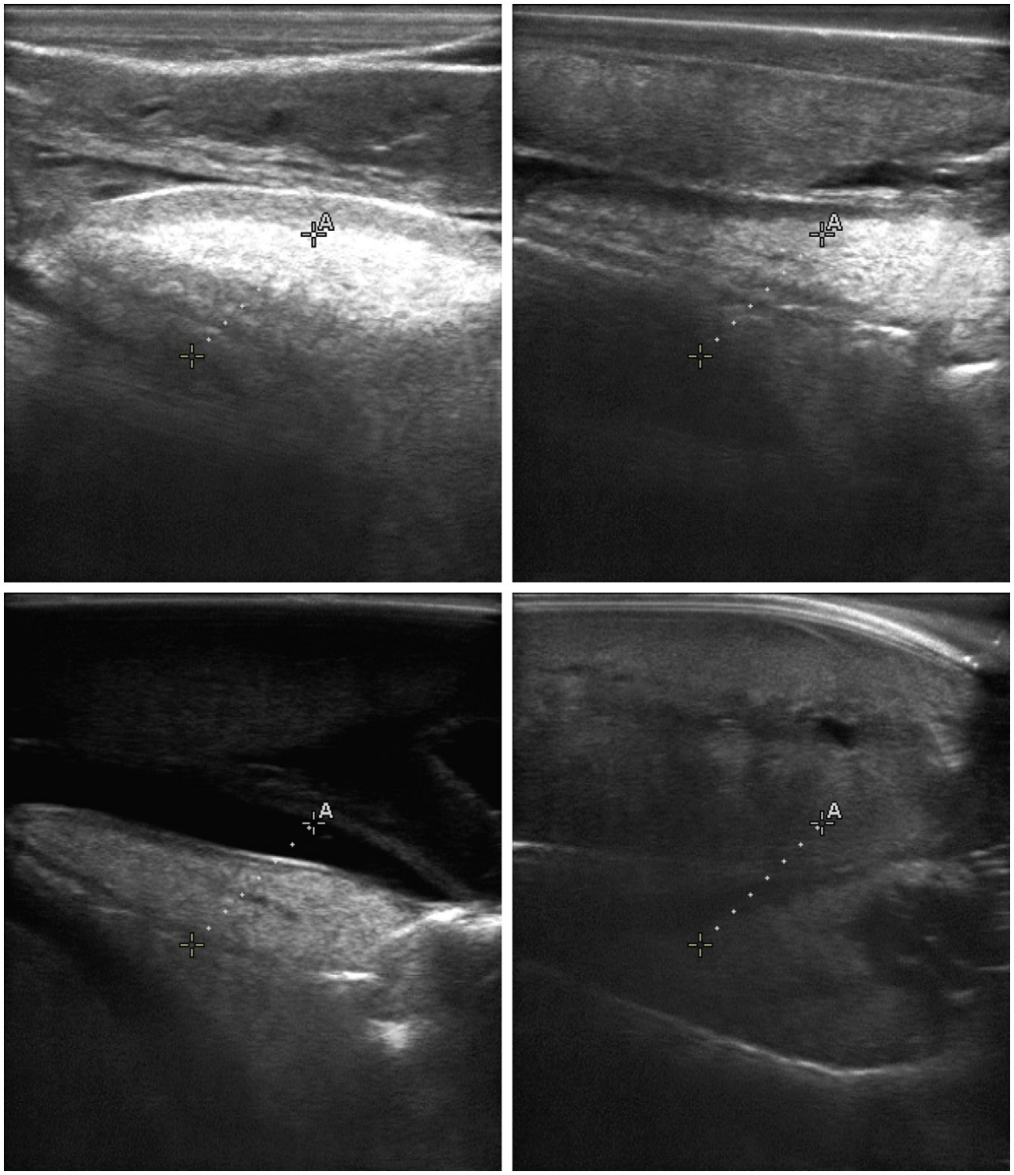
Examples of red abalone (*Haliotis rufescens*) ultrasound images excluded during quality filtering (Section 2.3.2). These images illustrate the three exclusion criteria: severe echoing artifacts producing duplicate or ghost structures (top row), absence of identifiable gonad and digestive gland anatomy (bottom right), and incomplete capture of the animal with the gonad region outside the field of view (bottom left). Filtering was performed by E.A.S., who had no prior training in abalone ultrasonography; only images with obvious defects were removed. Compare with the high-quality images shown in Figure 1.

**Figure 3:**
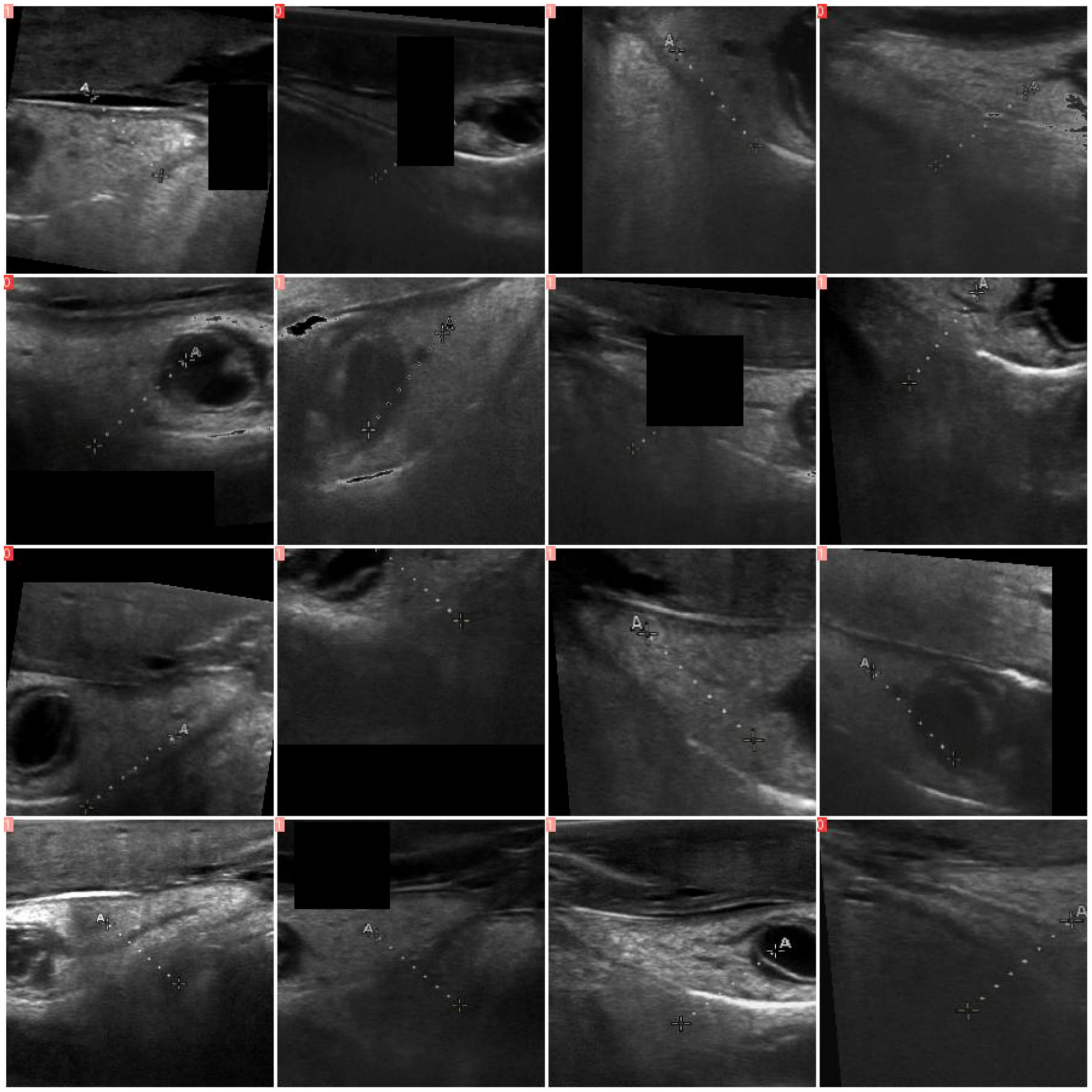
Examples of augmented red abalone (*Haliotis rufescens*) ultrasound images used during training. Augmentations applied according to YOLO defaults include random rectangle erasure (black regions), horizontal flipping, rotation up to 15 degrees, translation up to 10% of the image size, and brightness/contrast jittering. These transformations were applied uniformly across all model architectures to expand effective training diversity and reduce overfitting (Section 2.3.3).

Large abalone images were filtered for quality through visual inspection by E.A.S., who had no prior training in abalone ultrasonography. Images were excluded if they met any of the following criteria: (a) severe echoing artifacts in which the ultrasound beam reflected off the shell, creating duplicate or ghost structures; (b) absence of identifiable gonad and digestive gland anatomy; or (c) incomplete capture of the animal such that the field of view did not encompass the gonad region. Examples of excluded and retained images are shown in Figures 2, 3. Only images with obvious defects were removed; borderline cases were retained. This conservative threshold was chosen under the assumption that an operator with ultrasonography experience would filter more aggressively than an operator with none, and we therefore report results on a dataset that likely includes some suboptimal images. This filtering yielded a total of 246 high quality images from large individuals (Table 2).

**Table 2:** Distribution of large red abalone (*Haliotis rufescens*) ultrasound images by sex at each stage of preprocessing: unfiltered, quality-filtered, and augmented.

	Unfiltered	Filtered	Augmented
F	201 (22)	114 (22)	174 (22)
M	203 (25)	132 (22)	199 (22)
Total	404 (47)	246 (44)	373 (44)

Unique individual counts are shown in parentheses.

#### 2.3.3 Dataset partitioning

The final dataset consisted of only large individuals, from which 4 individuals of each sex were randomly assigned to the validation and test sets, respectively. The remaining 28 individuals (14 of each sex) were allocated to the training set. All images from a given individual were confined to a single partition, ensuring that no specimen appeared across multiple splits. Because the number of images per individual was uneven, the resulting proportion of total images in each partition did not correspond exactly to a 90/10 or 80/20 ratio; however, the individual-level constraint was maintained throughout. Best models were selected based on validation metrics, and the held-out test set remained untouched until final evaluation. No models were selected based on test set performance.

An initial stage of data augmentation was performed to generate synthetic data for balanced individual representation in the training split. In creating the synthetic data, existing images were rotated up to 15 degrees in either direction, random brightness and contrast values were jittered by 10%, and arbitrary zooming up to 20% (Figure 3) was applied with the torchvision 0.17.2 library (Paszke et al., 2019).

We note that an earlier iteration of the pipeline used image-level random subsampling on an 80/20 train/validation split without individual-based constraints. This approach was abandoned prior to any reported experiments due to data leakage concerns, and all results presented in this manuscript reflect the individual-based partitioning scheme described above.

### 2.4 Logistic regression on flattened abalone images

To classify abalone sex (female vs. male) from ultrasound images, we employed a logistic regression framework applied to flattened image representations. All analyses were implemented in Python v3.10 (Python Software Foundation, 2023).

Images were loaded and preprocessed using Pillow v10.2.0 (Clark, 2015). Each image was converted to grayscale and resized to 64 × 64 pixels, yielding a 4,096-dimensional feature vector per sample after flattening. The dataset partitions described above were maintained throughout all experiments, yielding training (*n* = 153 images; 80 F, 73 M), validation (*n* = 51 images; 22 F, 29 M), and held-out test (*n* = 42 images; 12 F, 30 M) splits. The corresponding individual counts for each split are reported in Table 3.

**Table 3:** Distribution of red abalone (*Haliotis rufescens*) ultrasound images by sex across training, validation, and held-out test splits for small and large size classes.

	Small			Large		
	Train	Val	Test	Train	Val	Test
F	201+121 (22)	40 (5)	39 (5)	80+60 (14)	22 (4)	12 (4)
M	170+96 (20)	30 (4)	29 (4)	73+67 (14)	29 (4)	30 (4)
Total	371+217 (42)	70 (9)	68 (9)	153+127 (28)	51 (8)	42 (8)

Cell values are formatted as raw_count+augmented_count(unique_individual_count), where the first value is the number of original images, the second is the number of synthetic augmented images, and the parenthetical is the number of unique individuals.

Pixel intensities were standardized to zero mean and unit variance using feature-wise statistics computed exclusively from the training set. Standardization and all subsequent modeling steps were performed with scikit-learn v1.7.2 (Pedregosa et al., 2011b). To reduce dimensionality and mitigate overfitting, principal component analysis (PCA) was applied to the standardized features. The number of retained PCA components was treated as a tunable hyperparameter, with candidate values of 5, 10, 20, 30, 50, 75, and 100. Array operations were handled with NumPy v2.2.2 (Harris et al., 2020).

Hyperparameter selection was performed via grid search over the regularization strength *C* (0.001, 0.01, 0.1, 0.5, 1.0, 3.0, 5.0, 7.0, 10.0, 15.0, 20.0, 50.0, 100.0), penalty type (L1, L2), solver (liblinear, saga), and the number of PCA components listed above. To account for class imbalance across the splits, inverse class-frequency weighting was applied during training via the class_weight=~balanced~ parameter in scikit-learn, which scales the loss contribution of each class in proportion to its representation in the training set. Model selection was guided by balanced accuracy on the validation set, which averages per-class recall and thereby prevents the selection of degenerate classifiers that predict only the majority class.

The best-performing configuration was serialized to disk using joblib v1.5.2 (Joblib Development Team, 2024). Final evaluation was then conducted on the held-out test set, which remained untouched during all tuning and model selection steps. We report accuracy, per-class precision, per-class recall, and per-class F1 score for each of the training, validation, and test partitions. A confusion matrix and cumulative PCA explained variance plot were generated using matplotlib v3.10.7 (Hunter, 2007).

This procedure was conducted independently on two versions of the training data: the original (non-augmented) images and an augmented variant. The augmented dataset was prepared separately and subjected to the identical training, validation, and test pipeline described above, preserving the same held-out test partition across both experiments to enable direct comparison.

### 2.5 CNN model architectures

Training was done using various classification models implemented in PyTorch v2.2.2 (Paszke et al., 2019). These models included VGG16, VGG19 (Simonyan and Zisserman, 2015), ResNet50, ResNet101 (He et al., 2016), and our own custom-built CNN models which inverted the structure of the Convolutional 2D layers and the structure of the 1D layers of the VGG architecture. YOLOv8 and YOLOv11 models were trained using the Ultralytics library v8.3.3 (Jocher et al., 2023; Jocher and Qiu, 2024), which provides a PyTorch-based training pipeline. All training was performed on NVIDIA A100 GPUs via Expanse at the San Diego Supercomputer Center and Bridges-2 at the Pittsburgh Supercomputing Center. Additional data augmentation on top of the original synthetic data generation was performed according to YOLO defaults for all models, which included random horizontal flipping with probability 0.5, color jittering with brightness, contrast, saturation, and hue variations of 40%, 70%, 70%, and hue factor of 0.015, respectively. For each image, a rectangle region was erased with probability 0.4 and size as the defaults, and a random translation of 10% of the image size was applied. Training was performed using binary categorical labels. Their respective output layers were set to a single sigmoid with the BinaryCrossEntropy loss function. Batch sizes were set to 48, 32, 16 and 128 for custom VGG, ResNet, Custom Reverse, and YOLO models respectively. Model checkpointing and early stopping was implemented using the validation loss (0.70 minimum) for the models respective loss function with initial monitoring after 100 epochs and a patience of 500 epochs.

Models were initially trained on small animals using the same procedures described above, but due to a lackluster performance, this approach was abandoned in favor of transfer learning, in which each architecture was initialized with the weights from its best-performing large-animal model and training was continued on the small animal image dataset (Table 3). The same individual-based partitioning, augmentation, and early stopping procedures were applied to the small animal splits.

### 2.6 Hyperparameter tuning

Custom CNN models were hyperparameters tuned across various learning rates, dropout rates, layer depth, layer breadth and optimizers. We applied various degrees of dropout in order to prevent overfitting. In total 22 model structures were trained on with varying learning rates, dropout rates and optimizers for a total of 44 different parameter combinations for each of the model structures, for a total of 968 custom CNN models.

### 2.7 Evaluation metrics

Models were evaluated based on training and validation losses as well as precision, recall, and accuracy. Models were chosen based on validation accuracy, and finally tested using our test dataset. We report only the best models, based on validation recall, precision, and accuracy, for each of the various architectures (Table 4).

**Table 4:** Best model performance by architecture for sex classification of large red abalone (*Haliotis rufescens*) ultrasound images, selected on validation metrics.

Model	**M Prec**.	**M Rec**.	**F Prec**.	**F Rec**.	**Train Acc**.	**Val Acc**.	**Test Acc**.
YOLOv8	0.905	0.845	0.816	0.899	0.961	**0.882**	**0.857**
YOLOv11	0.524	**0.952**	**0.917**	0.667	**0.996**	0.863	0.738
ResNet-50	0.667	0.615	0.833	0.862	0.811	0.824	0.786
ResNet-101	0.500	0.600	0.867	0.812	0.957	0.843	0.762
VGG-16	0.417	0.278	0.567	0.708	0.957	0.706	0.524
VGG-19	-	-	-	-	-	-	-
Custom-S	**0.917**	0.440	0.533	0.941	0.925	0.647	0.643
Custom-M	0.500	0.375	0.667	0.769	0.932	0.745	0.619
Custom-L	0.750	0.562	0.767	0.885	0.968	0.745	0.762
Reverse-S	0.417	0.417	0.767	0.767	0.900	0.686	0.667
Reverse-M	0.667	0.571	0.800	0.857	0.954	0.706	0.762
Reverse-L	**0.917**	0.579	0.733	**0.957**	0.993	0.784	0.786

Precision and recall are reported per class (M = male, F = female). Best values are shown in bold and second best are underlined. VGG-19 failed to converge to nontrivial results across all runs.

### 2.8 Feature extraction and visualization

We extracted the top five PCA components from images using the scikit-learn libraries in Python (Pedregosa et al., 2011a), and chose the top two components for K-means clustering. The optimal K clusters were determined using the elbow method using Within-Cluster Sum of Squares and the silhouette score. We also looked at plotting the third principal component against the first and second, as well as all top three using 3D matplotlib plotting libraries. The t-SNE dimensionality reduction technique (Maaten and Hinton, 2008) was used to visualize our model features using scikit-learn v1.5.0. Feature extraction was performed by extracting the weights of each of the best models and applying them to randomly subsampled images. Images were plotted using the OpenCV package.

### 2.9 EdgeAI device deployment

Using only our best models for edge device deployment, we present our best extra large YOLOv8 model. We exported the YOLOv8 model to ONNX format through Ultralytics export API which invokes torch.onnx.export. The ONNX model was then converted to a TensorRT engine using Ultralytics' onnx2engine utility with FP16 precision. The TensorRT engine was executed on a 8GB Jetson Orin Nano. Inference was made using a stream of prefiltered train and test images located in separate folders on the device.

## 3 Results

### 3.1 Data characteristics

Structure in the data was evaluated using filename-derived naming conventions, which allowed us to group images by individual identity, size, sex, and location. When individual identity was not considered and images were randomly split into training, validation, and test sets at the image level, the same animal could be represented in multiple sets, leading to biased training and overfitting. We found accuracy to be artificially inflated in images split without taking individuals into account. Once we enforced splitting by individual, model evaluation metrics were more in line with realistic performance, with initial validation and test accuracy at approximately 60%. These analyses indicated that a higher proportion of images from small animals were misclassified than those from large animals. We decided to split the data into large and small size classes, and initial training and validation results for large animals improved with metrics reaching approximately 70%, whereas models trained on small individuals remained near 55%. We continued to explore outliers and observed that images that were not clear, had imaging artifacts, or did not show clear gonads and digestive glands were often misclassified across various models. We then labeled them as poor-quality images, and manually removed them from training, we provide an example in Figure 2. This led to improved model performance and metrics.

#### 3.1.1 K-means clustering

We applied K-means clustering to CNN-extracted ultrasound images features after reducing dimensionality using PCA, retaining the top three components. Cluster numbers from K = 2 to 9 were tested. The elbow method and silhouette analysis both indicated K = 2 as the optimal number of clusters (Supplementary Figure S1). Our PCA scatter plots (Supplementary Figure S2) showed overlap among large and small males and females, with no clear separation by sex or size.

### 3.2 Model performance comparison

As a baseline, we trained a logistic regression classifier on PCA-reduced flattened image features using the same individual-based data splits (Section 2.4). Without data augmentation, this resulted in 100%, 51%, and 71% accuracy in train, validation and test image sets, respectively, with the train-to-test gap indicating clear overfitting to the low-dimensional feature space. With data augmentation, accuracy was 67%, 51%, and 76% in train, validation and test image sets, respectively, partially mitigating overfitting but remaining near chance on the validation set. These results are consistent with the PCA scatter plots (Supplementary Figure S3), which show substantial class overlap. These results establish a non-deep-learning baseline against which all CNN architectures in Table 4 should be compared.

We then benchmarked CNN architectures, beginning with VGG and ResNet, followed by our reversed custom CNN. Our implementations of VGG and ResNet models resulted in the models quickly over-fitting. Dropout was added to our VGG and custom CNN models to prevent overfitting.

Our VGG16 and VGG19 models are identical to their original implementation with the exception of adding dropout. We performed hyper-parameter tuning across optimization functions, learning rates, batch size, activation functions and dropout rates. This resulted in 95.7%, 70.6% and 52.4% accuracy in train, validation and test image sets, respectively.

**Figure 4:**
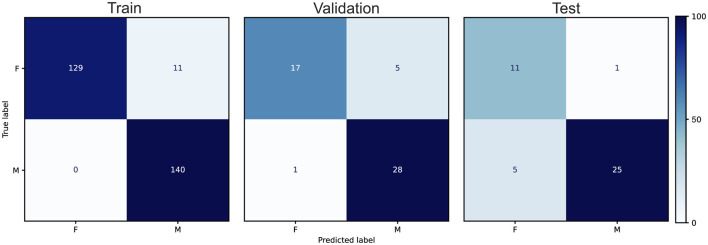
Confusion matrices for the best-performing YOLOv8 model across training, validation, and held-out test splits for sex classification of large red abalone (*Haliotis rufescens*). Rows represent ground truth labels (F = female, M = male) and columns represent predicted labels. Cell values are raw image counts; color intensity indicates the percentage of each true class assigned to each predicted class. The test set confusion matrix shows 11 of 12 female images and 25 of 30 male images correctly classified, corresponding to 85.7% overall test accuracy.

During systematic exploration of architectural variants, we evaluated an inverted VGG model in which the layer channel progression was reversed, starting with 512 channels and progressively reducing to 64, in both the 2D and 1D layer blocks. Regularization via dropout layers was implemented after each block. This resulted in better performance than the vanilla VGG models and was on par with some of our ResNet models, resulting in 99.3%, 78.4% and 78.6% (Reverse Large) accuracy in train, validation and test image sets, respectively. Our ResNet models performed better than our custom VGG, and custom CNN models. Our Resnet 101 achieved 95.7%, 84.3% and 76.2% accuracy in train, validation and test image sets, respectively. Our final implementation of the image classification models, YOLOv8x-cls (Jocher et al., 2023), resulted in 96.1%, 88.2% and 85.7% (X-Large) accuracy in the train, validation, and test sets, respectively (Figure 4). We also tested a newer implementation of YOLO11x-cls which resulted in 99.6%, 86.3%, and 73.8% accuracy, respectively.

### 3.3 Feature analysis

**Figure 5:**
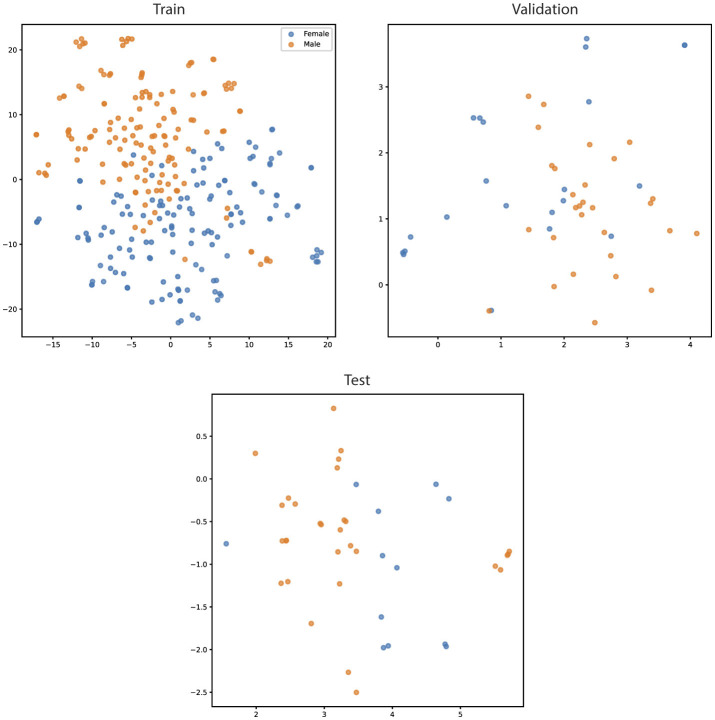
Two-dimensional t-SNE projections of intermediate features extracted from the best-performing YOLOv8 model for large red abalone (*Haliotis rufescens*) ultrasound images, shown separately for training, validation, and held-out test splits. Each point represents a single image, colored by ground truth sex label (blue = female, orange = male). Substantial class overlap is visible in the training and validation projections despite high classification accuracy (96.1% and 88.2%, respectively), indicating that the learned decision boundary operates in a high-dimensional space that t-SNE does not preserve in two dimensions. Silhouette scores on the intermediate features were low (train: 0.096, validation: 0.096, test: 0.080), consistent with the visual overlap and suggesting that sex discrimination relies on subtle, distributed features rather than well-separated clusters.

**Figure 6:**
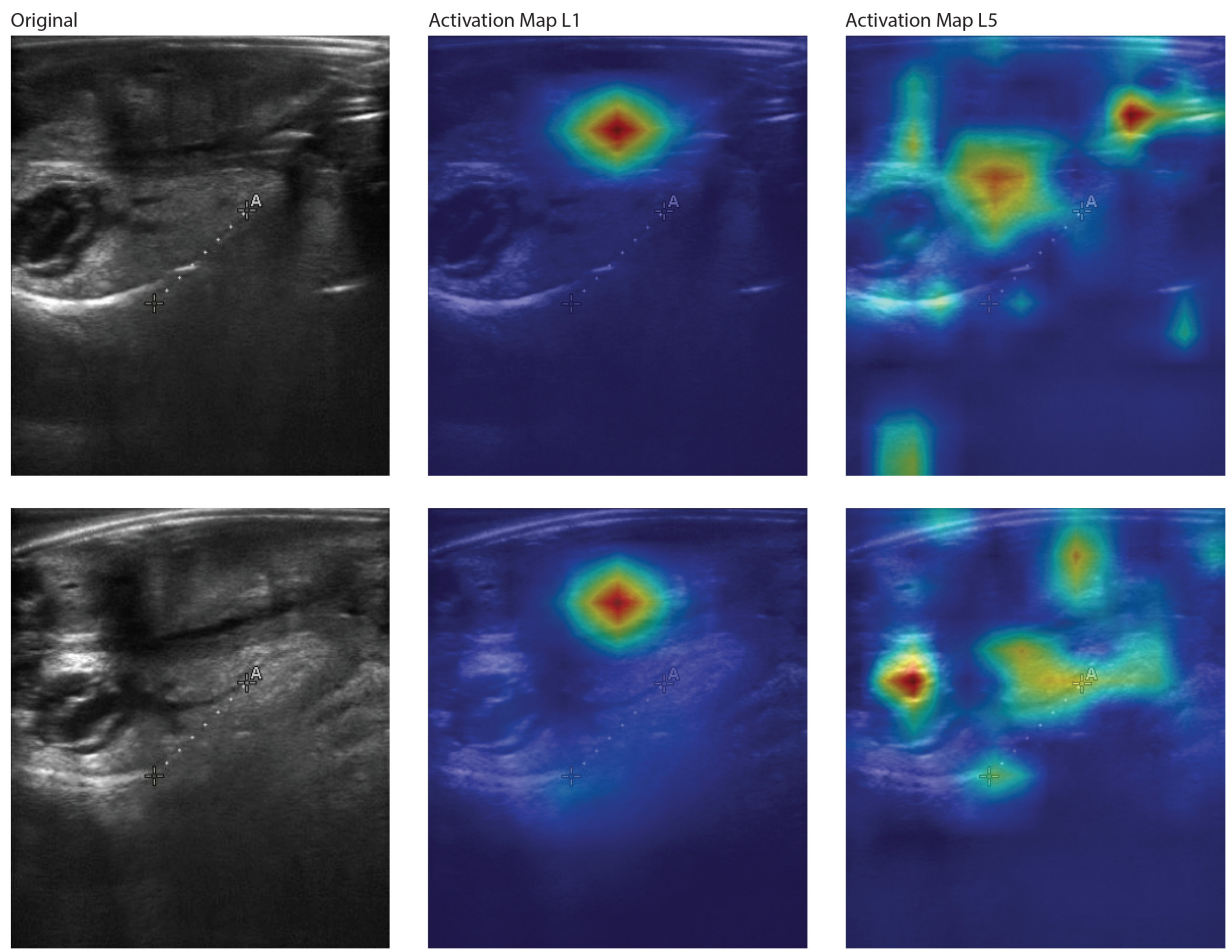
Feature activation maps from the best-performing YOLOv8 model for two correctly classified large female red abalone (*Haliotis rufescens*) images. Each row shows the original ultrasound image (**left**), the first-layer activation map (L1, **middle**), and the fifth-layer activation map (L5, **right**). Activation intensity follows a gradient from dark purple (lowest) through blue, green, and yellow to red (highest). At L1, activation is concentrated in a single focal region coinciding with the gonad. At L5, activation distributes into multiple smaller foci along the gonad and the boundary between the gonad and the digestive gland, suggesting that deeper layers resolve finer anatomical distinctions rather than responding to a single dominant region.

**Figure 7:**
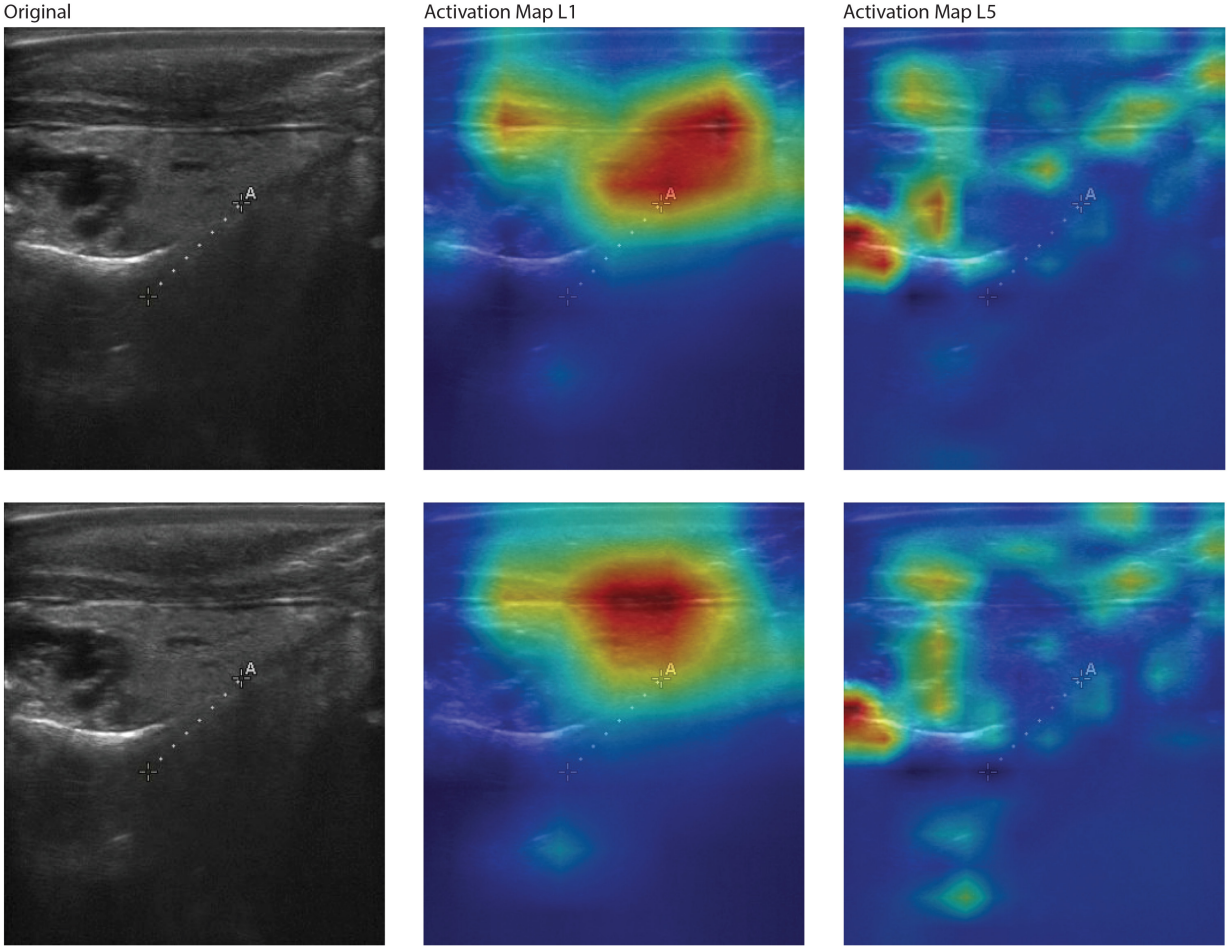
Feature activation maps from the best-performing YOLOv8 model for two correctly classified large male red abalone (*Haliotis rufescens*) images. Each row shows the original ultrasound image (**left**), the first-layer activation map (L1, **middle**), and the fifth-layer activation map (L5, **right**). Activation intensity follows a gradient from dark purple (lowest) through blue, green, and yellow to red (highest). At L1, activation covers a broad region spanning the gonad and extending into adjacent tissue. At L5, activation redistributes into multiple smaller foci, including the lower gonad boundary and areas below the gonad. This pattern is consistent with the female activation maps (Figure 6): early layers respond to large-scale tissue structure while deeper layers resolve spatially distinct anatomical features.

**Figure 8:**
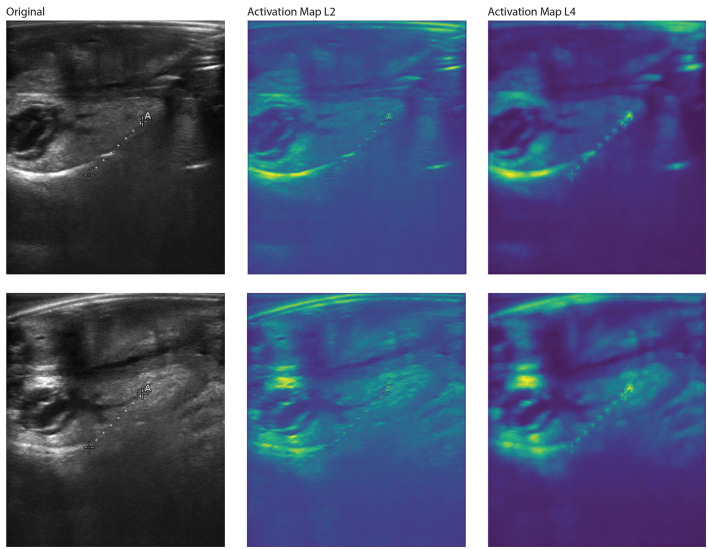
Feature activation maps from the Reverse-L VGG model for two correctly classified large female red abalone (*Haliotis rufescens*) images. Each row shows the original ultrasound image (**left**), the second-layer activation map (L2, **middle**), and the fourth-layer activation map (L4, **right**). This model uses a viridis-like colorscale with activation intensity ranging from dark purple (lowest) through blue and green to yellow (highest); red is absent, indicating different color scales of the activation compared to the YOLOv8 maps (Figure 6). At L2, the highest activation coincides with the gonad region, but notable activation also appears on the “A” annotation marker and the measurement line. At L4, activation persists on the gonad but also appears at the top of the image where echoing artifacts are present, indicating that the Reverse VGG model partially attends to non-anatomical features. This contrast with the YOLOv8 activation maps, which focus more exclusively on gonadal tissue, is consistent with the lower classification accuracy of the Reverse VGG architecture.

**Figure 9:**
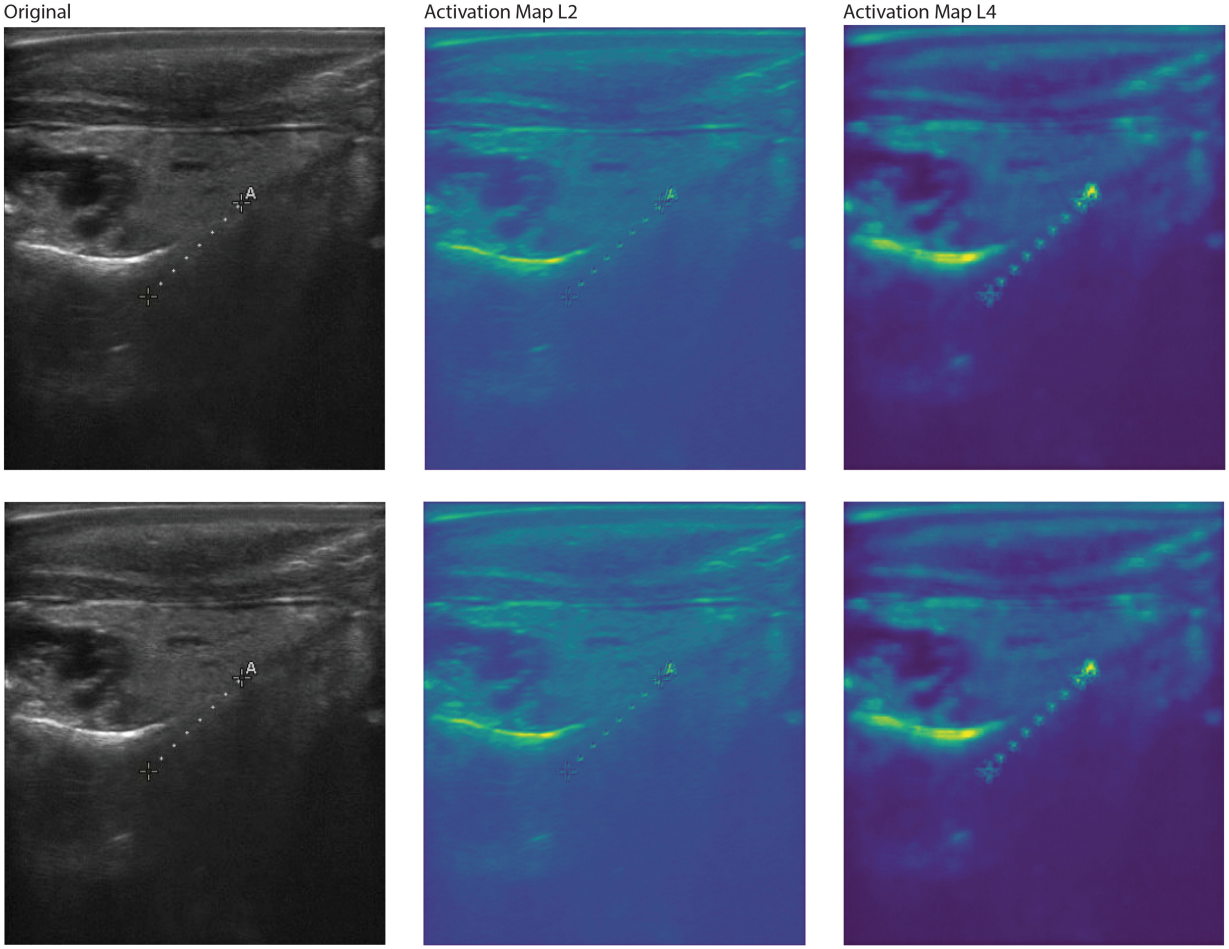
Feature activation maps from the Reverse-L VGG model for two correctly classified large male red abalone (*Haliotis rufescens*) images. Each row shows the original ultrasound image (**left**), the second-layer activation map (L2, **middle**), and the fourth-layer activation map (L4, **right**). Colorscale follows the same viridis-like gradient as Figure 8, from dark purple (lowest) to yellow (highest). At both L2 and L4, the strongest activation is concentrated along the gonad band. At L4, additional activation appears on the “A” annotation marker and measurement line, consistent with the artifact attention observed in the female Reverse VGG maps (Figure 8). The persistence of activation on non-anatomical features across both sexes reinforces the conclusion that the Reverse VGG architecture, while outperforming standard VGG, is more susceptible to imaging artifacts than YOLOv8.

The t-SNE results from the YOLOv8 model illustrate some separation of male and female large abalone, as well as some that do not cluster together (Figure 5). We performed various feature extraction maps and visualized them (Figures 6–9). We can observe multiple activations across the gonad tissue for most of our best models, as well as activation in adjacent anatomical areas. As we progress through the layers, we continue to see activation of the gonad and a deactivation of the digestive gland, until the feature maps become too abstracted to interpret.

### 3.4 EdgeAI device inference

Inference times averaged 14.50ms per frame, but also required pre-processing and post-processing for a total of 1.35ms on the Jetson Orin Nano and a test accuracy of 85.7%.

## 4 Discussion

### 4.1 Principal findings

In this study, we present, to our knowledge, the first application of machine learning to automate sex classification in abalone using non-invasive ultrasound imaging technology. By developing an ultrasound image dataset for red abalone and benchmarking a logistic regression baseline alongside a suite of convolutional neural network architectures, we show that CNN models such as YOLOv8 and ResNet can distinguish between male and female gonadal morphology from soft tissue ultrasound images for this species, whereas linear classifiers on PCA-reduced flattened image features cannot. These results indicate that ultrasound based image classification in combination with machine learning technology can provide a practical, non-lethal tool for sex determination in both conservation and production aquaculture settings.

Our results highlight several practical insights for future deployment: (1) accurate model evaluation requires data partitioning by individual rather than by image to avoid artificially inflated performance; (2) image quality strongly influences classification accuracy, with low-contrast or artifact-laden scans contributing disproportionately to errors; and (3) size-dependent anatomical variation can bias model predictions, necessitating size-aware sampling or stratification. Although overall performance is promising, particularly for large individuals and high-quality images, the variability in model confidence underscores the need for continued dataset expansion, improved standardization of imaging protocols, and integration of automated quality control.

We initially explored various image filters such as non-local means denoising which is meant to improve ultrasound details by removing white speckle noise using different methods such as Gaussian blurs, but ultimately did not use these methods for our final models, as it did not improve model performance. Initial data preprocessing yielded deceptively good results, but the similarity of ultrasound images belonging to the same individuals meant that the naive splitting by image caused data leakages throughout the dataset, so the final preprocessing allocated splits by individuals instead. Additionally, the quality of small abalone ultrasounds were far inferior to those of the large abalone, and due to the nature of the small dataset at hand, we settled in favor of high-quality images only and relied on heavy data augmentation and dropout to prevent the overfitting of our models.

### 4.2 Architecture insights

The removal of small abalone images and the final filtering of low-quality large abalone ultrasounds raised model performance from near-random guessing accuracy of 60% toward the 80% mark, a significant performance increase. Our implementation of VGG models, despite strong data augmentation with YOLO presets, generally either failed to converge or performed worse than our YOLO models. We propose that due to the model age and lack of features present in models like ResNet with its skip connections or the lightweight and modular design of YOLO, training with VGG was unstable and inefficient due to the large parameter count. Since the VGG family are particularly large models fitted with the classification task of a tiny training set of 280 images with few options for regularization, it fell behind the other models consistently. This was also most likely due to how the VGG architecture starts with a low number of features but then expands into a larger number of features, doubling at each layer block (64 → 128 → 256 → 512 → 512). We reasoned that this expansion pattern, while effective for natural images, may be suboptimal for ultrasound data.

In standard VGG, early layers with few channels are tasked with extracting low-level features such as edges and textures, which then serve as building blocks for increasingly abstract representations in deeper layers. However, ultrasound images contain pervasive speckle noise that manifests as high-frequency texture patterns across the image. When VGG's early layers, constrained to only 64 channels, attempt to encode these patterns, they may allocate limited representational capacity to noise artifacts rather than biologically meaningful features. These noisy low-level representations then propagate through the network, potentially corrupting the higher-level features built upon them. In contrast, our reverse architecture begins with a large number of channels (512), allowing the model to capture a diverse set of features before progressively compressing them (512 → 256 → 128 → 64), which we propose forces the network to retain only the most discriminative information while discarding speckle-related noise. This design philosophy aligns with the Information Bottleneck principle (Tishby and Zaslavsky, 2015), which posits that optimal representations compress input information while preserving task-relevant features, precisely what our architecture enforces structurally through simultaneous channel and spatial reduction.

In response to the poor performance of the VGG model family, we additionally implemented various architectural changes as spin-offs of the VGG architecture, only modifying the depth and breadth of the layers, but performance remained similar to that of the VGG models. We also tested the Reverse-S/M/L family of models, obtained by literally reversing the convolutional layers of the VGG architecture and fine tuning the depth and breadth of each layer, starting with a large channel count and gradually reducing it over the layers (512 → 256 → 128 → 64). Counterintuitively, the models performed better than the VGG, and modified VGG models. CNNs are generally designed with inductive-biases toward detecting low level features first, gradually connecting them with long-range dependencies later on in the model. However, with each variant of the reverse models outperforming its forward counterparts by approximately 5% on average, it is very likely that this architectural change helps in the abalone ultrasound domain. Notably, recent work by (Liu et al. 2025) systematically evaluated filter placement topologies in ResNet-based architectures and introduced “RevNet” (Reverse ResNet), which implements the same decreasing filter configuration (512 → 256 → 128 → 64). Their findings demonstrated that this contrarian strategy, reducing filters by half across successive layers, can achieve performance on par with, or superior to, the conventional approach on standard benchmarks. Our results extend this finding to the ultrasound imaging domain, representing, to our knowledge, the first application of this inverted channel topology to marine invertebrate tissue classification.

First, the grainy noise in ultrasound imaging hurts conventional CNN models by introducing a large number of insignificant local artifacts. These small-scale details, such as speckle patterns, can dominate the low-level feature extraction process. On the other hand, the reverse model starts by encoding a wide variety of features, and the gradual reduction of channels over time forces the model to compress the information and wipe out unmeaningful small edge details. Second, compression of channels in the reverse model aids in reducing overfitting, particularly in settings with limited data. With a small dataset, it is very easy for standard CNNs to learn to rely on minute details like speckles in the ultrasound data, which are actually meaningless patterns. We see this in the failure of convergence in the VGG-19, which is a particularly large and monolithic model that significantly overfits on the training data. On the other hand, the reverse family of models is able to move past the noise. Third, our architecture implements what we term “double compression”: the combination of decreasing channel depth (512 → 256 → 128 → 64) with max pooling at each block creates simultaneous channel and spatial reduction. This is architecturally distinct from encoder-decoder networks such as SegNet (Badrinarayanan et al., 2017), which also reduce channel depth in their decoder pathway but do so while upsampling spatial resolution for segmentation tasks. In contrast, our architecture continues to downsample spatially while reducing channels, creating an aggressive information bottleneck optimized for classification rather than spatial reconstruction. This double compression forces the network to retain only the most salient biological features, such as gonad and digestive gland morphology, while discarding both stochastic speckle noise and fine-grained spatial details that do not contribute to sex discrimination. Fourth, we placed dropout at the end of the convolutional block, immediately before flattening. This strategic placement, combined with the high initial channel count (512 filters), provides regularization at the critical transition between spatial feature extraction and dense classification. The dropout layer acts as an additional noise filter, randomly zeroing activations that may have encoded speckle-specific patterns during training, thereby encouraging the network to learn robust representations of anatomically meaningful structures.

However, the ResNet model family still remained competitive with the reverse models. We propose that both models were able to combat overfitting, but did so in different ways. The ResNet poses several advantages over the VGG models, namely through their key skip connections, improving gradient flow and stabilizing training. The ResNet models enable and encourage identity layers, so that noisy layers can be nullified and skipped over via the skip connections, and is in general more suitable to a robust variety of applications. Thus the ResNet models also do not need a large dataset to efficiently generalize, and are generally able to combat noisy environments seen in ultrasound data.

Though we did not explore a combination of the reverse layers and the ResNet family (adding skip connections to the reverse CNN models), this represents a promising direction. Recent work by (Liu et al. 2025) demonstrated that their RevNet architecture, which combines the reverse filter topology with ResNet's residual block framework, achieved competitive or superior performance on standard benchmarks. A hybrid architecture combining our inverted channel progression (512 → 64) with skip connections could potentially leverage both the noise-filtering properties of progressive channel compression and the gradient flow benefits of residual connections, representing a logical next step for ultrasound classification tasks.

Our final and best performing models come from the YOLO family. We trained the YOLOv8x-cls and YOLOv11x-cls models from scratch, meaning we randomly initialized the model parameters/weights, instead of loading pretrained weights. At the time of this writing, the YOLOv8 models outperformed the YOLOv11 models, but we expect this may change as updates continue to be made to YOLOv11.

### 4.3 t-distributed Stochastic Neighbor Embedding (t-SNE) analysis

We first discuss the qualitative outputs of t-SNE on our dataset. Surprisingly, the most separated set of data belonged to the testing set, despite it having the lowest accuracy. There is significant overlap in the training and validation t-SNE plots, which is particularly surprising for such high accuracy results observed in our final YOLOv8 model. This suggests that the decision boundary is complex and high-dimensional, and has a very fine line for distinguishability. The model successfully separates classes in the original high-dimensional space, even though t-SNE fails to visualize this separation in 2D. This suggests that the learned features are simply still extremely complex. To further confirm this result, we found that the train, validation, and test silhouette scores were 0.0964, 0.0963, and 0.0795, respectively, on the intermediate feature values.

First, the intermediate features do not form well-separated clusters. This makes it particularly difficult for t-SNE to produce a well-separated graph, and it likely means that the decision boundaries are extremely fine-grained and subtle. Second, it aligns with human intuition: even trained experts find this classification task difficult without explicit visual cues. The model must rely on subtle and distributed visual signals, rather than clearly defined or localized features. Third, this task results in signals being distributed across features. In particular, the final features contain a variety of features which all have strong signals, as opposed to few neurons containing almost all of the discriminating features. This suggests that a variety of properties contribute together toward determining the output; possibly features such as gonad shape, brightness, and texture cues which provide only subtle hints and must work together.

### 4.4 Practical applications

*In vivo* sex determination in abalone is typically conducted via visual inspection, which can be unreliable due to gonad maturation states, yet our image classification models also identify structural features in the ultrasound images that are not apparent to the human eye. The activation of areas adjacent to the gonad raises the question of whether the proportion between the gonad and the digestive gland area is also important for sex determination. This capacity to highlight anatomically relevant structures—potentially as a consequence of the aggressive information bottleneck created by simultaneous channel and spatial reduction—suggests that our architecture may be particularly well-suited to soft tissue classification, given more optimization, in ultrasound where texture and regional morphology carry discriminative information. Although some of the models we trained with are over a decade old, they still perform well and above random chance when updated to meet current standards via more recent data augmentation, image preprocessing and regularization methods.

By coupling ultrasonography with machine learning-based inference on edge computing devices, this framework lays the groundwork for real-time, operator-independent sexing tools that can enhance spawning efficiency, reduce handling stress, and support scaling of recovery and sustainable propagation efforts for endangered abalone. For abalone programs where the number of available individuals is large, the ability to determine sex with accuracies above 80%, as observed for large red abalone after filtering low quality images, represents a substantial labor savings compared with manual evaluation of every animal, particularly when hundreds of individuals can be screened per hour with manual checks reserved for only a subset of animals or uncertain classifications. In practice, operators acquire multiple ultrasound frames per animal under the standardized tank conditions used in hatchery and aquaculture facilities; the envisioned deployment workflow would automatically screen frames for quality, classify sex on passing frames, and flag low-confidence predictions for manual review, reducing dependence on any single image being artifact-free. For captive breeding programs for threatened and endangered species, where broodstock numbers are limited and many individuals are immature, rapid non-lethal screening can be used to identify the relatively few animals that have reached reproductive maturity and prioritize them for spawning.

As future models are extended from sex classification to also approximate gonad maturity, we expect additional gains in spawning advancement, larval recruitment, and juvenile survival. Studies using ultrasonography in abalone have shown that individuals with higher gonad indices or greater gonad relative average thickness exhibit higher total and relative fecundity, higher fertilization and hatching rates, lower larval abnormality, and higher larval attachment than low index individuals, directly linking gonad development to reproductive performance and larval quality (Zou et al., 2025). Together with work demonstrating that ultrasound-derived gonad indices can be used to track maturation and time spawning events in red abalone and endangered California abalone species, these findings suggest that incorporating maturity prediction into machine learning models could further improve broodstock selection. In particular, preferentially selecting females with larger and more developed ovaries is likely to select for more mature ova, important for non-feeding, free-swimming larvae that depend entirely on maternal nutrient reserves. Improving our ability to identify and use such females should translate into improved larvae quality and recruitment outcomes in both conservation and production hatchery programs.

### 4.5 Limitations

Several limitations warrant consideration. First, the dataset consisted of only 34 large size class individuals, limiting statistical power and potentially inflating performance estimates. Second, models trained directly on small abalone performed poorly, and although transfer learning from large-animal weights improved test accuracy from approximately 55% to 60% (Supplementary Table S1), this remained insufficient for reliable classification. This suggests size-dependent morphological variation that was not captured, or perhaps their size limited the quality of the ultrasound on smaller animals leading to a larger amount of reflection and artifacts. Third, image quality significantly impacted classification accuracy; a substantial proportion of images were excluded due to artifacts, and we acknowledge that our current models were developed and evaluated using quality-filtered images from large individuals. Performance under variable imaging conditions and with smaller animals remains to be established. However, the intended deployment environments for this technology are conservation hatcheries and commercial aquaculture facilities, where abalone are imaged while submerged in tanks under standardized conditions, not in unpredictable field settings. The imaging protocol used in this study was performed at the breeding facility under the same conditions that would apply in routine hatchery operations. In practice, operators acquire multiple frames per animal, and we envision a deployment pipeline in which automated quality screening precedes sex classification (Section 4.6), with uncertain classifications flagged for manual review. This multi-frame, quality-aware workflow would substantially reduce dependence on individual image quality. Fourth, the study focused on a single species; transferability to endangered species (*H. cracherodii, H. sorenseni*) requires validation and significant time and resources. Fifth, the binary classification task does not address reproductive maturity assessment, which remains an important goal for spawning management and an interesting and natural follow up to this study.

### 4.6 Future directions

We identify five areas for future development. The first is expansion to additional species, particularly endangered abalone where this technology would have immediate conservation value. The second is integration of reproductive maturity assessment, potentially through multitask learning that jointly predicts sex and gonad development stages. The third is development of automated image quality control. Manual filtering in this study was performed by an operator with no prior ultrasonography training, demonstrating that the criteria are accessible, but the process remains subjective and would benefit from standardization. Deep learning-based quality assessment has been demonstrated for cardiac ultrasound (Abdi et al., 2017) and high-frequency dermatological ultrasound (Kucharski et al., 2022), and a similar approach could automate the identification of artifact-laden or incomplete abalone ultrasound frames prior to sex classification. The computational cost of such a quality filter is minimal and compatible with the edge inference hardware described in Section 3.4, enabling a two-stage pipeline in which quality screening and sex classification run sequentially on a single device. The fourth is collection of larger datasets to enable more robust generalization across size classes and imaging conditions. The fifth is architectural exploration combining our inverted channel topology with residual connections; recent work by (Liu et al. 2025) demonstrated that their RevNet (not ResNet) architecture, which applies this decreasing filter pattern (512 → 256 → 128 → 64) within ResNet's residual framework, achieves competitive performance on standard benchmarks, suggesting that a hybrid approach could leverage both the noise-filtering properties of progressive compression and the gradient flow benefits of skip connections for ultrasound classification tasks.

Ultimately, our results demonstrate that computer vision models can classify red abalone sex from ultrasound images with useful accuracy, even when trained on a limited dataset. Although additional data are needed to improve robustness across size classes and image quality, the present models already achieve inference speeds that are compatible with on site use on embedded EdgeAI devices such as the Jetson Orin Nano, allowing biologists to perform multiple scans and classify animals in real time. As larger and more diverse ultrasound datasets are collected and practitioners become more familiar with AI embedded systems for classification, we expect both accuracy and reliability to improve, supporting more efficient broodstock management and spawning decisions in conservation and production aquaculture and increasing the throughput of endangered species rescue and breeding efforts.

## 5 Conclusions

We demonstrate that convolutional neural networks can classify red abalone sex from ultrasound images with 85.7% accuracy on held-out test data, establishing the feasibility of automated, non-invasive sex determination for this species. The YOLOv8 architecture outperformed classical CNN models (VGG, ResNet) and custom architectures, likely due to its efficient handling of limited training data. Notably, our finding that a reverse channel architecture (512 → 256 → 128 → 64) outperforms standard VGG on ultrasound data suggests that progressive channel compression may help CNNs overcome speckle noise, a hypothesis consistent with the Information Bottleneck principle (Tishby and Zaslavsky, 2015) and recently corroborated by (Liu et al. 2025), who demonstrated competitive performance with decreasing filter topologies on standard benchmarks. Our architecture implements what we term “double compression”: simultaneous channel reduction and spatial downsampling via max pooling, which is architecturally distinct from encoder-decoder networks like SegNet that reduce channels while upsampling spatially. This double compression, combined with strategic dropout placement at the end of the convolutional block, appears to enable the network to attend to biologically meaningful features such as gonad and digestive gland morphology rather than speckle artifacts, as evidenced by our feature activation maps. To our knowledge, this represents the first application of an inverted channel architecture with double compression to marine invertebrate tissue classification from ultrasound imaging, and the first demonstration of automated sex determination in abalone from ultrasound. By coupling ultrasonography with edge-deployable inference, this framework enables real-time, operator-independent sexing tools for both conservation breeding programs and commercial aquaculture. Future work should expand to endangered species, integrate reproductive maturity assessment, and explore hybrid architectures combining our inverted topology with residual connections.

## Data Availability

Source code is available in the following GitHub repositories, https://github.com/ESB-AI-Lab/AbaloneClassification, https://github.com/ESB-AI-Lab/red-abalone-sex-classification. The first link contains finalized code for the final training done for this manuscript, and the second link contains code for initial exploratory data analysis and training prior to finalizing a standard pipeline to be used across all model architectures. The raw data supporting the conclusions of this article will be made available by the authors, without undue reservation.
