# Supplementary material for: Deep learning classification of reproductive tissue from ultrasound: sex determination in red abalone (Haliotis rufescens)
Source: Front Artif Intell. 2026 May 18;9:1794183. doi: 10.3389/frai.2026.1794183 (PMC13223049; doi:10.3389/frai.2026.1794183)
Supplement: Supplementary file 1 [file Data_Sheet_1.pdf]

Supplementary Material

1 SUPPLEMENTARY TABLES AND FIGURES

1.1 Tables

Model	M Prec.	M Rec.	F Prec.	F Rec.	Train Acc.	Val Acc.	Test Acc.
Large YOLOv8	0.905	0.845	0.816	0.899	0.961	0.882	0.857
Small YOLOv8	0.647	0.733	0.778	0.700	1.000	0.714	0.603

Table S1. Evaluation metrics for small animals using transfer learning of our best from trained on large animals using YoloV8. Model precision, recall, and accuracy. Best values are bolded, and second best values are underlined. VGG-19 completely failed to converge to nontrivial results across its runs.

1.2 Figures

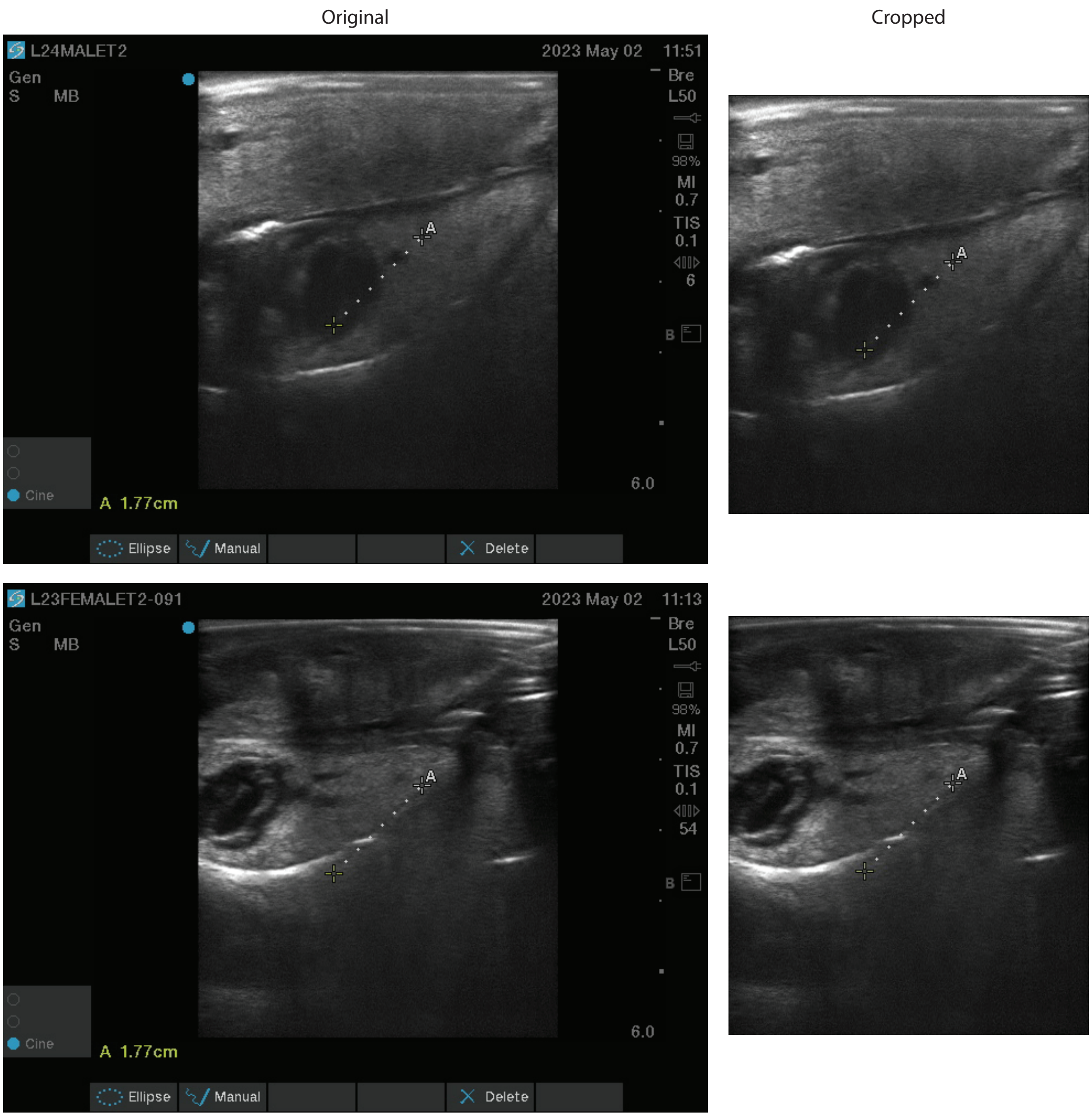

Figure S1. Examples of automated cropping of red abalone (*Haliotis rufescens*) ultrasound images. Each row shows one individual, with the original image (left) and the autocropped result (right). The original images contain system overlays including the individual identifier and sex label in the filename (e.g., “L24MALET2”, “L23FEMALET2”), acquisition date, transducer parameters, and measurement annotations. Retaining these overlays would allow models to classify sex from text rather than from gonadal morphology. The automated cropping pipeline (Section 2.3.1) removes these overlays, retaining only the raw ultrasonography region.

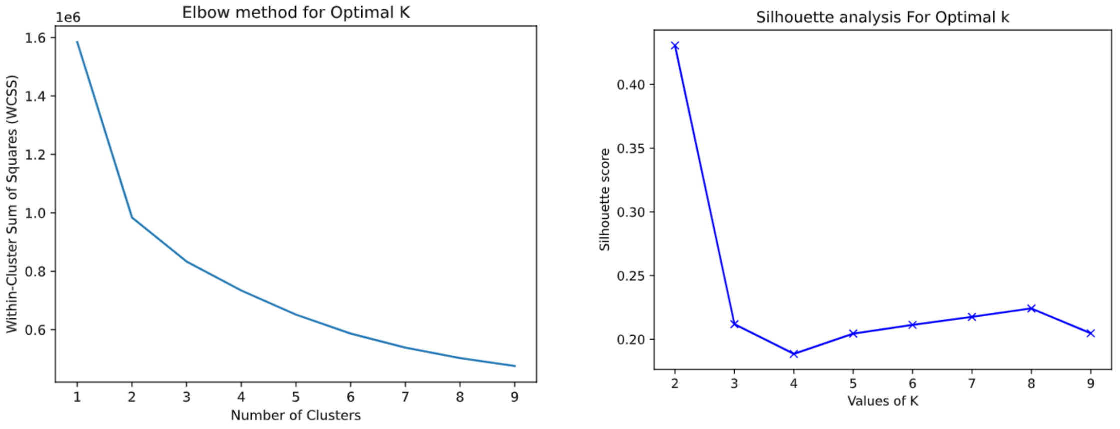

Figure S2. Cluster number selection for K-means clustering of red abalone (*Haliotis rufescens*) ultrasound image features. (Left) Elbow method: Within-Cluster Sum of Squares (WCSS) as a function of K, showing a distinct inflection at K = 2. (Right) Silhouette analysis: the highest silhouette score (approximately 0.43) occurs at K = 2, with scores dropping sharply for $K \geq 3$. Both methods converge on K = 2 as the optimal cluster number; however, PCA scatter plots (Figure S3) show substantial overlap between sexes within these two clusters, indicating that the clustering structure does not correspond to a clean male–female separation.

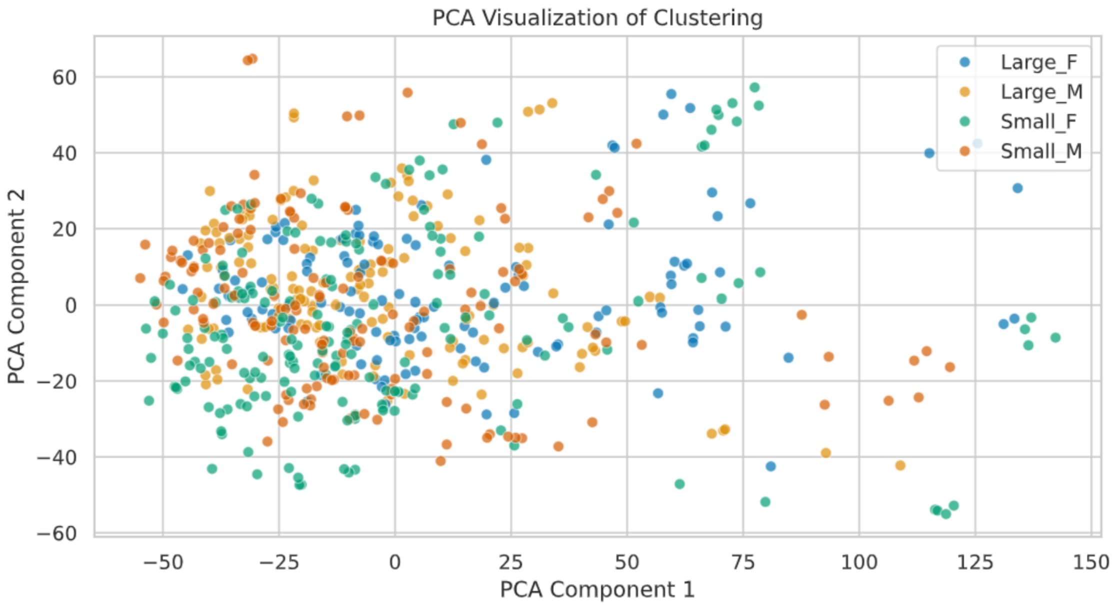

Figure S3. Two-dimensional PCA projection of red abalone (*Haliotis rufescens*) ultrasound image features colored by sex and size class (Large_F = large female, Large_M = large male, Small_F = small female, Small_M = small male). The first two principal components show extensive overlap among all four groups, with no clear separation by sex or size. This overlap is consistent with the near-chance accuracy of logistic regression on PCA-reduced features (Section 3.2) and supports the conclusion that sex discrimination in abalone ultrasound images requires non-linear feature extraction, as achieved by the CNN architectures evaluated in this study.
